# Including highly educated migrants in academia to improve their health—protocol for a pilot intervention

**DOI:** 10.3389/fpubh.2024.1347992

**Published:** 2024-10-28

**Authors:** Khadra Yasien Ahmed, Lars T. Fadnes, Bernadette Kumar, Wegdan Hasha, Esperanza Diaz

**Affiliations:** ^1^Department of Global Public Health and Primary Care, University of Bergen, Bergen, Norway; ^2^Bergen Addiction Research, Department of Addiction Medicine, Haukeland University Hospital, Bergen, Norway; ^3^Division for Health Services Research, Norwegian Institute of Public Health, Oslo, Norway; ^4^Department of Biomedicine, University of Bergen, Bergen, Norway; ^5^Oral Health Center of Expertise in Western Norway, Bergen, Norway; ^6^Department of Health and Functioning, Western Norway University of Applied Sciences, Bergen, Norway

**Keywords:** highly educated migrants, Norwegian academia, integration, self-rated health, health professionals

## Abstract

**Introduction:**

Norway’s healthcare system needs a diversified work force to meet societal demands for improved cultural competence. However, many migrants in Norway who were educated as health professions in their home countries are not practicing these professions. This may negatively affect their physical and mental health and hinder their personal social integration. Though good health is often seen as a precondition for work, relevant working activities can also improve health. However, including health professionals with foreign education in academic institutions prior to receiving necessary accreditation is a complex task. This study will pilot an intervention aiming to improve health through meaningful integration of these professionals in academic environments.

**Materials and methods:**

This paper is a protocol for a non-randomized pilot intervention study targeting migrants who are waiting for their health education accreditation in Norway. To test the benefits of meaningful activity on health and explore possibilities for implementing such activity, we have designed a six-month long intervention consisting of including nurses, doctors, and other highly educated migrants with healthcare backgrounds between 20 and 67 years of age, into health-related working tasks, at two higher education institutions in Bergen, Norway. The intervention will be tailored according to the participant’s expertise. This hybrid type 2 pilot protocol paper will present how feasibility, fidelity, dose received (satisfaction), and dose of exposure (participation), will be assessed and whether the intervention is experienced as beneficial for the participants’ health as primary outcome utilizing both quantitative and qualitative methods.

**Conclusion:**

We present a complex, personalized intervention that has the potential for large scale implementation in the future. By thoroughly presenting our designed intervention and assessment methods, this protocol will add to the study’s transparency and facilitate replicability and comparison with future studies. This study will be of benefit to the migrants themselves, policy makers, government agencies and academia at large as it can point to a unique and sustainable way of speeding up the integration of highly educated migrants in their respective fields in a new host country.

## 1 Introduction

In Norway, 42% of migrants, compared with 14% of the majority population, are overqualified for their current jobs, and a substantial number of these migrants have a health professional background (1). Simultaneously, Norway and other high-income countries are experiencing a shortage of healthcare professionals, in addition to an aging population that will pose further stress to the healthcare system (2). High-income countries are also becoming increasingly diverse with respect to migrant populations, thereby diversifying needs among the citizens (3). A multicultural workforce has been suggested as one possible solution for this challenge (2).

Immigrants in Norway, defined as persons born abroad of two foreign-born parents and four foreign-born grandparents, comprises 18.9% of the total population (4, 5). In the period from 2016 to 2020, the proportion of employed people with an immigration background increased from 15.7 to 18.1% (5). There were approximately 34,400 people with a foreign background who worked within the health and social services in Norway in 2014. Among them, 10,300 were nurses, while nearly 5,100 were doctors. Norway’s doctor coverage is 5.2 doctors pr. 1,000 residents for the years 2021 (2, 3). Even with this increase in labor among people with an immigration background, there is a growing need for health personnel in Norway, and recruiting professionals from abroad has been suggested as a solution (3).

Implementing strategies to improve workforce diversity and workplace inclusivity is highly lauded, but also difficult to execute. Intentions to diversify the workplace and workforce have failed due to lack of implementation strategies and sustainable solutions in academia and numerous other industries (6). Many reasons for these findings have been proposed, but viewing an increase in diversity as a short-term measure has emerged as one key explanation (6–11). Most high-income countries prioritize the integration of migrants with diverse skillsets into the labor market (12). Until recently, research on integration efforts has focused on migrants with low educational attainment and are struggling in the workplace (10). There is still limited literature regarding highly educated migrants with health education backgrounds (10, 13–18).

Health professionals can be understood as a group of professionals that “maintain health in humans through the application of the principles and procedures of evidence-based medicine and caring. Health professionals study, diagnose, treat and prevent human illness, injury and other physical and mental impairments in accordance with the needs of the populations they serve” according to the WHO definition of the term (19). Typically, studies on integration include health only as a prerequisite for integration (20). However, bureaucratic bottlenecks that stall the healthcare sector’s accreditation process may negatively affect the physical and mental health, as well as social integration of highly educated migrants with healthcare backgrounds (21–24). The integration paradox highlights this complexity, as migrants with higher education feel less connected to their host countries due to their perceived relative deprivation compared to the host population (25). High and unmet expectations also fuel a sense of disengagement from the host country. A lower sense of belonging is further increased by a higher sense of perceived discrimination (10). Furthermore, migrants’ poor integration is costly for the host society due to productivity loss (17, 18).

As health and integration are intertwined (24), we hypothesize that by including migrants in meaningful activities, we will see improvement in self-rated health (SRH) and quality of life (QoL). SRH is used as a proxy for general health, well-known from the literature and related to several other health outcomes and death (26). Meaningful integration can be understood as the process in which a person utilizes his/her experiences, competence, and resources professionally and in other life domains. Health, meaningful integration, and working life do not exist in silos, but rather in mutual interaction whereby each influences the others (9, 10). Integration through meaningful activities is positive for health, yet there is a gap in the literature whether this is also the case for migrants with higher health education (8, 10).

This intervention is part of the “Integration for health “(Int4Health)-study, which emerges from needs detected during the previous CHART-study (Changing Health and health care needs Along the Syrian Refugees’ Trajectories to Norway). The CHART-study studied health, health care needs, and quality of life among Syrian refugees during migration and their first year after arrival in Norway (27). In that study, migrants from Syria were studied pre- and post-arrival to Norway. Findings detected that SRH and QoL improved with greater time spent in Norway (27). The current study expands beyond Syrian refugees to all migrants, with a specific focus on health professionals.

To our knowledge, no studies have been conducted on the links between meaningful integration and health among highly educated migrants. Furthermore, if such a link exists, we need information on how meaningful integration activities can be facilitated in academic institutions. To close these research gaps, we have designed a pilot study to test the effect on health and the feasibility of an intervention including highly educated migrants with healthcare backgrounds in meaningful, work-related tasks in academic institutions. In this paper, we present the pilot study’s protocol. In this paper, we present the pilot study’s protocol.

## 2 Methods and analysis

### 2.1 Study design

This protocol presents a pilot study for a non-randomized intervention in the city of Bergen, Norway, with a control arm in the city of Kristiansand, Norway. This pilot intervention implements an effectiveness-implementation hybrid design, known as a hybrid type 2 trial. Such designs study both effectiveness – the degree to which an intervention is successful in producing the desired result – and the impact of the implementation strategy on the outcomes – the methods used to achieve the results. In these studies, outcome measures and hypotheses are clearly pre-defined. The implementation strategy resembles what would be intended for future implementation in a definitive randomized controlled trial. A hybrid type 2 trial design therefore allows our study an initial assessment of the intervention’s effect on the participant’s health and to explore which components of the implementation strategy need further refinement by studying its feasibility, fidelity, dose received (satisfaction) and dose of exposure (participation) through qualitative and quantitative methods. For pilots, hybrid type 2 trials are useful especially if no full-scale intervention is executed (28–30).

### 2.2 Setting

This study’s intervention was developed at the Faculty of Medicine at the University of Bergen, in collaboration with the Western Norway University of Applied Sciences, both of which will host the study participants. The participants will therefore have their placements at either of these locations. The project team collaborates closely with the Norwegian Labor and Welfare Administration (NAV) and Bergen municipality. These two stakeholders are important because they are formally in charge of migrant integration programs, and because many of the potential participants are already engaged in NAV programs, which also provide them with financial support. Following an agreement with NAV in Bergen, participation in this intervention will not result in any loss of income for participants. Kristiansand is our control group is due to our formal cooperation with Kristiansand municipality in the study that allowed us to include Kongsgård School Center as place to conduct the surveys for the participants in the introductory programs there.

### 2.3 Timeframe

Meetings prior to the intervention with relevant stakeholders such as NAV, Bergen municipality, non-governmental organizations and user representatives were conducted in the spring of 2022. Content for the preparation days for the participants were also developed during spring of 2022. Recruitment for the pilot study started in November 2022 and will last until June 2023. To recruit enough participants and mentors, different participants will be recruited and be enrolled in two rounds, prior to each of the intervention periods lasting 6 months. The first wave of piloting is planned from January to June 2023 while the second wave of the piloting will last from August to December 2023. Analyses of the data gathered through the intervention, publication of papers and dissemination to stakeholders and society will start in 2024 and last until the end of 2025.

### 2.4 User involvement in developing the intervention

Two user representatives, one male and one female, both of whom are pharmacists and have migrant backgrounds from Asia and the Middle East respectively, were consulted about recruitment strategies and several practical subjects related to the intervention. They provided insight into challenges the target group may face regarding work hours and childcare, as well as the necessity of properly distributed and appropriate information. For the selection of the mentors and their involvement in the project, we consulted with an academic staff with experience in inclusive teaching environments at the Faculty of Medicine. The first author and several senior researchers in the team of the “Integration for health” research project have migrant background, which is an additional asset for recruiting participants and organizing the fieldwork logistics and has been successful.

### 2.5 Recruitment of participants

To recruit suitable participants, formal letters are sent to the heads of social services along with NAV who conveyed the information to their social workers. When the social workers receive this information, they can contact us for more information to clarify the adequacy of the intervention for a particular client and/or provide us the contact information for consenting clients. In addition to the explained collaboration with NAV for the recruitment of participants, flyers, leaflets, and information sheets about the study will be distributed to non-governmental organizations and churches in Bergen. Information will also be sent by email to stakeholders in migration, health, education, and civil society. Various migrant interest groups will be contacted and visited. Dissemination on Facebook in expatriate forums and associations of foreign doctors and dentists of Norway will also be utilized.

Once identified, each participant’s professional background is assessed by going through his/her curriculum vitae. The participant is thereafter interviewed for 30 min by the first author to intake further information regarding their place of living, education, work, Norwegian proficiency level, and aspirations for the coming 6 months. Information about the intervention is then provided and participants who satisfy all inclusion criteria and do not meet any exclusion criteria, as explained below, are eligible to enroll in the pilot study.

To recruit mentors, a formal letter will be sent to the dean of the Faculty of Medicine in Bergen along with all five heads of Departments at the medical faculty to convey information about the study. The same letter will also be sent to the heads of health education at the Western Norway University of Applied Sciences, and all heads of Departments at The Medical Faculty at the University of Bergen. We request that they disseminate the information to their various scientific environments and offering the possibility of a visit by a member of the research team for further details. In addition, information will be issued on the Departments’ intranet pages so the staff can access this information directly.

Once the participants have been recruited, the University of Bergen’s and the Western Norway University of Applied Sciences’ search engines and personal knowledge of the research team is used to match specialists in various health fields to the participants´ professional backgrounds. Once participant-mentor matches are identified, the participant’s curriculum vitae is sent along to the potential mentor with information about the study; if necessary, we will follow up with these potential mentors. Compatibility between mentor and mentee is assessed based on professional interests, work experience, and formal education to the best of our ability. As soon as the mentor agrees to join, a meeting with the mentee is arranged to introduce the pair and clarify the scope of tasks and time spent at the institution. The entire recruiting process, including contacts with stakeholders, will be repeated for the two rounds of different participants to make the steps as similar as possible. However, we also acknowledge that we would not be able to match everyone perfectly. In those few cases, we informed both parties that whoever they were matched with, was not a full match, but that we would do our best to facilitate if challenges arise.

Regarding recruitment in Kristiansand, the school administration coordinated the practical and logistical challenges such as rooms, time slots, and information for the students and teachers. Both the school’s management and teachers received a formal letter prior to the recruitment. Only those students who met the inclusion criteria were instructed by the teachers and attended the survey. Participants in the control group were informed by the school’s psychosocial team before our arrival about the various mental health support systems in Kristiansand and encouraged to contact the local health care services if need be.

In Kristiansand we recruited participants with a variety of educational backgrounds and not only with health background as in Bergen. This was due to recruitment challenges, as finding individuals with such a specific background was practically difficult and would have given us a smaller pool of candidates and thus less statistical power. However, we acknowledge that some baselines characteristic like years of residence or level or integration scores can differ, therefore describing and eventually adjusting for the differences will be crucial. The reason for having Kristiansand as a control group is due to earlier collaboration in the CHART-study. Their Introduction program, at the time of developing the protocol, was one of the few agencies that worked for early integration of refugees in the labor market in Kristiansand.

### 2.6 Intervention

The intervention for a single participant consists of a six-month assistantship with a personal mentor, matched as explained above, at one of two higher education institutions in Bergen. Through their mentors, the mentees are integrated into the larger educational and research groups at the institutions. The participants in the intervention will first take part in 3 days of preparation for the intervention. Training components include an overall introduction to the study and the educational institutions involved and discussions on challenges that may arise and how to solve them as well as training in cultural competence. They will also sign the informed consent form and answer the survey described below, for the first time.

**Figure 1:**
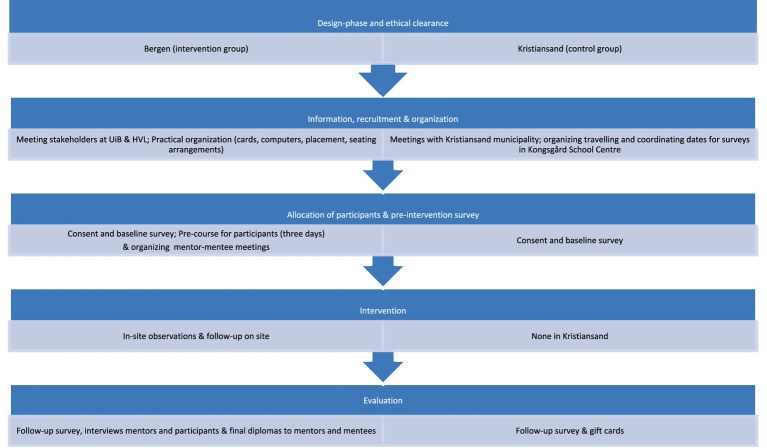
Flow chart overview of the intervention.

They will then attend their institutions for a minimum of 2 days per week, taking part in their mentor’s or research group’s daily activities, which may entail meetings, supervision, lectures, and other relevant educational activities with an increasing degree of independence. We will not provide cultural competence training to the mentors, but we will encourage the mentors to utilize and incorporate the participants’ cultural competence at the working place. The mentors will be the primary source of information for the participants at each placement site. The mentor’s role will consist of, among other tasks, to organize a working space, introduce the participants to the other colleagues, include the participants in tailored activities, involve the participant in the group’s daily activities and generally support the participants throughout the period (Figure 1).

### 2.7 Eligibility criteria

We recruit migrants defined as persons born outside of Norway who have a residence permit in the country, living in Bergen (intervention) or Kristiansand (controls). Their Norwegian language proficiency must be at level A2 or above. To be eligible for the intervention group in Bergen, it is mandatory that participants have a health professional education gained abroad but lack full accreditation to work in the healthcare field in Norway. Participants in Kristiansand must have at least a high school education level to be included, but do not require a health professional background. Participants in Bergen must commit to the intervention for 6 months.

### 2.8 Exclusion criteria

Participants with physical or mental ailments that require extensive medical follow-up will be excluded from the intervention (e.g., they appear to suffer from deep depression). If participants are already involved in meaningful, health-related work like researching in health care areas, they will also be excluded from the study. Participants with a high level of psychological distress in the intervention group (assessed with Hopkins Symptom Check List with 10 items with mean item score of >1.85) will be referred to a psychologist, who will assess if the participants need further follow-up.

### 2.9 Data collection and measurement of effect

To the best of our knowledge, this is the first study to pilot an intervention for highly educated migrants with a main outcome of self-reported health improvement. However, there are no single formulae to assess complex intervention studies, with the intervention tailored to each participant. Thus, different holistic outcomes for health measures are included in the survey. Additionally, to assess possibilities for future implementation of the intervention and the impact of the implementation strategy on intervention outcomes, a complementary mixed methods approach will be used (31) using interviews and observations to collect and assess data on feasibility, fidelity, dose received (satisfaction), and dose of exposure (participation) (28). Quantitative information will be collected via questionnaires for the intervention and control groups. The intervention arm will also undergo personal interviews and observations. Lastly, mentors will also be interviewed.

#### 2.9.1 Questionnaire

We have developed a questionnaire that all participants will answer twice: in Bergen, participants will complete the questionnaire once pre- and once post-intervention, and participants in the control arm in Kristiansand will also complete the questionnaire twice but with a six-month period between the responses.

The questionnaire is available in Norwegian and English and includes 40 questions with an expected time to complete of 35 min (Supplementary material A). The following questions, which have been used in several cultural settings, will be included in the survey (32–35).

Sociodemographic, sense of coherence and integration: Background questions on sociodemographic and migration factors are adopted from the CHART-study (26, 27). These questions include information about arrival year, emigration reason, educational level and background, marital status, language level, and household members.

The sense of coherence scale with 13 items (SOC-13) is used to measure subjective sense of comprehensibility, manageability, and meaningfulness in one’s life. SOC-13 assesses how people view their life and identifies how they use their resistance resources to maintain and develop good overall health (36–38). Our questionnaire includes 10 questions from the Immigration Policy Lab Integration Index (IPL-12) related to the domains of integration, linguistic integration, and psychological integration (38). Two questions related to income level and understanding of political matters were excluded as they were not deemed to be necessary.

Variables on health and wellbeing: Self-rated health (SRH) is the individual’s own evaluation and perception of their health and is widely used in research related to health disparities in diverse populations. In our study, self-rated health is the main health outcome, assessed by the question: “How do you currently assess your health?” with the possible answers “very poor,” “poor,” “none,” “good,” or “very good.” Furthermore, the question “Do you suffer from long-term (1-year) illness or injury of a physical or psychological nature that impairs your daily life?” and if yes, “How would you describe the impairment in terms of slight moderate or severe?” is included from the Nord-Trøndelag Health Study-3 (HUNT-3) but is not the main outcome (39).

The General Health Questionnaire (GHQ-12) is used to assess general mental health. The GHQ-12 consists of 12 items, each one assessing the severity of a mental problem over the past few weeks using a 4-point Likert-type scale from 0 to 3. The total score ranges from 0 to 36 (27). Individuals with scores above the cut-off point of 12 could be classified as possibly having mental health problems associated with depression, anxiety, somatic symptoms, and social dysfunction (40, 41). The Hopkins Symptom Cheklist-10 (HSCL-10) measures symptoms of anxiety and depression. The HSCL-10 is comprised of ten questions asking the respondent to rate the burden of various symptoms of anxiety and depression during the last week on a 4-point Likert scale with range from 1 to 4 (42). A score ≥ 1.85 is considered a valid cut-off value for prediction of mental distress (27).

Quality of life (QoL) is a broad concept covering all aspects of life, including health and non-health-related elements, that is appropriate to use when studying how exposure impacts physical, psychological, and social well-being. WHO-5 Well-Being Index will be used to measure subjective psychological wellbeing during the last 2 weeks (43, 44). The raw score is calculated by totaling all five answers. The raw score ranges from 0 to 25 and to obtain a percentage score ranging from 0 to 100, the raw score is multiplied by 4. Using a WHO-5 cut-off score of ≤50 is recommendable when screening for clinical depression (41). In addition, we use three more questions from HUNT-3 (39).

#### 2.9.2 Qualitative data

Outcome data related to the implementation strategy and effectiveness of the intervention will also be qualitatively collected through interviews and observation (28).

Interviews: To gain a deeper understanding of the experiences of the mentors and mentees, both parties will be interviewed at the end of the intervention. There is no interview component for the control group participants. The interviews will include questions related to the role of the mentorship, the experience of the mentee, and the perceived effect on the institutions. Mentees will also be asked to reflect on the intervention’s effectiveness on their health and lives. The interviews will follow a semi-structured interview guide (Supplementary material B); they will be recorded and transcribed verbatim.

In terms of design, we will apply the convergent parallel design as we will collect qualitative and quantitative data simultaneously, but we will analyze the data sets separately. The aim of this design is that the data sets will enrich and inform each other. A thematic approach alongside an interpretative phenomenological analysis (IPA) will be used in the qualitative analyses (38, 45). The IPA method will give us access to the lived experiences of the participants and mentors. Data will be analyzed with an inductive-deductive approach. Our theoretical background for this paper will be theories on meaningful integration and health (38, 46, 47). Theories on feasibility and fidelity, implementation, and evaluation will also be applied. The first author will conduct the initial analysis of the data, supervised by the last author. Once this is completed the analyses will be reviewed and modified together with the coauthors (48).

Observations: The first author will conduct systematic observations following a pre-structured checklist (Supplementary material C) for all participants in the intervention group during their placements. The observations will be used to assess the scope of tasks carried out by the participants and the fidelity of the implementation strategy. Each participant will be observed at least once in their working environment and observations will last between 30 and 60 min. A complete overview of the methods and measures of the pilot study is presented in Table 1.

**Table 1:** Data collection and evaluation method outcomes.

Evaluation method	Evaluation outcome measures
Questionnaire	Self-rated health and other health outcomes, sociodemographic- and migration-related factors
Observations	Fidelity, feasibility, dose of exposure
Individual interviews: participants	Experienced differences on self-rated health and other outcomes, dose received (satisfaction), dose of exposure (participation)
Individual interviews: mentors	Effect on institutions, feasibility, fidelity, dose of exposure (participation)

### 2.10 Power calculation and sample size

This pilot aims to evaluate both the feasibility and effectiveness of the piloted intervention. However, the sample size will probably not be sufficient to rely on statistical significance to address the effectiveness in health outcomes. For reporting the results of the pilot study regarding effectiveness, we will therefore look at trends by using relative risk measures with CI without relying on statistical significance only. If the study is found to be feasible and the trends of effectiveness in a positive direction for the participants, a new study involving a full evaluation of the effect on health as measured by the SRH will require a total sample size of 120 participants with 40 participants in the intervention arm – 20 for each round – and 80 participants in the control arm, based on the assumptions of a 1:2 individually randomized controlled trial with superiority design, with 80% power, 0.05 significance level of assessment, and standard deviation of 1.0 for both arms. The minimum detectable difference between the two arms is calculated to be 0.55.

### 2.11 Statistical analysis

Sociodemographic- and migration-related variables measured at baseline will be described for both the intervention and control arms. Continuous variables will be described with mean, standard deviation, and interquartile range. Categorical variables will be described as counts and percentages (absolute or relative frequency).

We will conduct intention to treat (ITT) analyses. However, it might not always be feasible to obtain information from participants who withdraw from a six-months intervention. As a part of the feasibility exploration, we will try to contact these participants by phone after the intervention period. This will allow us to compare the baselines assessments for those who left the intervention and see if they differ from the rest of the participants.

The effect on health will be measured quantitively regarding changes in self-perceived health for each subject between the intervention and control participants. Based on the questionnaire, SRH scores will be calculated pre- and post-intervention/non-intervention for each participant in both arms. Mean SRH scores will be calculated in both arms and for both time points. Differences in the mean SRH scores at the two time points will be calculated to measure changes in SRH over time. Any difference in SRH change between the two arms will be estimated using both crude (unadjusted) analysis and linear (mixed) regression. We will explore changes in other health and wellbeing assessments in a similar way to determine whether a different measure better detects changes in health. However, we will obtain basic sociodemographic and migration related factors at the baselines questionnaire that will allow us to describe the participants for each of the rounds. In addition, we will collect data on perceived changes in health through qualitative interviews.

Assuming that covariates at baseline are mostly complete, missing outcome values at follow-up due to drop-outs (lost to follow-up) from the study, will be tried corrected for, depending on missingness mechanism, using either simple approaches such as linear regression (Assuming Covariate-Dependent Missing Completely at Random) or more complex approaches [assuming missing at random (MAR)], such as GEE together with multiple imputation or inverse weighting, or using (linear) mixed model approach (using maximum likelihood methods) (49).

### 2.12 Evaluation of implementation strategy

Evaluating an implementation strategy is a complex endeavor for which there is not consensually accepted guidelines (28, 50). Many evaluating components can be included, but in this case, we will focus on feasibility, fidelity, dose received, or satisfaction, and dose of exposure, or participation, as suggested in the guidelines for conducting feasibility and pilot studies for implementation trials (28). In short data on evaluation will be collected through interviews and observations of both participants and mentors following a structured schema for observations and semi-structured interview guides for mentors and participants.

*Feasibility* will be assessed in our study by asking both mentors and participants how they experienced the implementation of the intervention, including the challenges they faced. For mentors, we will ask questions related to the execution of the mentor role. For participants, feasibility will be assessed by asking about the challenges and facilitators related to the placement, both at the personal level and regarding the institutions where they were placed. In addition, feasibility will be assessed through observations at the field.

*Fidelity* will be assessed through observations in the field compared to the study protocol by reviewing our implementation strategy, assessing intervention delivery, and evaluating how the implementation was in line with the purpose of the study and its aims (51).

*Dose received* (satisfaction) is defined as the participants’ satisfaction with the dose of the intervention received and will be assessed through questions to participants regarding the scope of tasks they executed and to which degree they value them in both professional and personal terms (52).

*Dose of exposure* (participation) is defined as the extent to which the participant actively engaged with, interacted with, and was receptive to the intervention. It will be assessed by asking the participants and the mentors about their stay at the institution, the activities they participated in, and their total time spent at the institution.

## 3 Discussion

Integration for health (Int4Health) is a research study anchored in the need to address a societal challenge. This protocol is a part of Int4Health aiming to describe how a pilot intervention aiming to improve health through meaningful professional integration, can be implemented. Executing interventions in academia is both complex and multifaceted, with various factors outside of the researcher’s control that can impact delivery of the intervention. Therefore, we must prioritize thorough planning and execution of the intervention. Protocols are also important for transparency, evaluations, and future replications of findings (53). For these reasons, we present in this paper the protocol of a hybrid type 2 study testing an intervention aimed to improve migrant health through meaningful activities.

The possible challenges to the present pilot study must be acknowledged, nonetheless. First, a non-randomized intervention could be vulnerable to unmeasured, confounding variables. Further, differences between the intervention and control arms’ locations and inclusion criteria (e.g., education level) might compromise comparability of the groups. A possible solution for both challenges could have been implementation of a waiting-list design; however, given that our participants already face the issue of waiting for extended periods for work accreditation in their professional field, adding an additional period of waiting was not considered an acceptable alternative.

Next, we acknowledge that the pilot’s small sample size will not have power and design to evaluate full effects on health, and if the pilot is found feasible and experienced positively, it must be later evaluated in a randomized controlled trial design. Recruitment is time-consuming when the inclusion criteria are strict, and the participants must commit to the intervention for at least 6 months. To address this, we have planned two rounds of recruitment and will only include the numbers that our partners believe to be feasible within the pilot period. To further address the small sample size, our methods include both questionnaires and interviews asking participants for their thoughts on how they perceived the intervention’s effect on their health so that we can assess nuances in the intervention’s impact. Reaching out twice 6 months apart to the same control participants in Kristiansand may also pose challenges to data collection, as participants may move, change phone numbers, or avoid contact with the researchers. Though having a control arm is a strength, this arm is unlikely to be a complete match for the intervention arm. Despite these challenges, the control group can still provide us with valuable insights on the development of health in a comparable time period regarding migrants in a compulsory educational setting.

Furthermore, various recruitment challenges may arise before, during, and after recruitment. Loss to follow-up and lack of participants is always a challenge when conducting interventions of this kind. To prepare for a future, full-scale study evaluating the intervention’s effects, we considered that recruiting up to 40 participants for the pilot would give us the necessary information to prepare for larger-scale recruitment. Closely following up on the participants in each round may also strengthen commitment to staying in the program. The observations of the participants on site along with midway evaluation is key to maintain engagement and address challenges that may arise. Therefore, the close relationship between the participants and the first author may function as a buffer against drop-out and is a key strategy for participant retention in both rounds. Given that this is a research study limited in time, we will not have time for long term evaluation of the participants which is a limitation. Nonetheless it would be desirable to contact them 1 or 3 years after the intervention when the project is upscaled.

Accessing the right mentors is also likely to post a significant challenge due to the limited availability of mentors, who will not receive extra resources for participating. Some mentors may also need special accreditation from both the head of their institute and head of administration before allowing anyone into certain workspaces (e.g., laboratory). We intend to use our networks and send information early on to heads of institutes to proactively prevent these problems.

Another challenge is balancing the duration of the intervention. On the one side, 6 months may be too short of a period to detect quantitative changes in health. On the other side, participants may feel that the six-month intervention period is already too long, given that participation is unpaid and does not aid them with the formal accreditation process. However, participation in the study may be a step towards relevant paid employment through gaining a professional network and better understanding of the system.

Lastly, the first author’s combined role as coordinator, administrator, and researcher may be a weakness in this study, as this may contribute to bias, without any blinding. Developing close professional relationships between the first author and participants could also create a certain level of bias in the data, primarily in interviews. Among the measures to counteract this is to thoroughly work on reflexivity, ask colleagues to conduct a portion of the interviews, and include the whole research team in analyzing the anonymized data.

Participation in the intervention is low-risk and we anticipate few adverse effects. Some participants may experience only short-term health and integration improvements. Other participants may experience worsened mental and somatic health after completing the intervention. If this happens, we will guide the participant to the right health care provider.

## 4 Ethics and dissemination

Participants in both arms will be provided with participant information sheets designed in compliance with the Declaration of Helsinki ethical research standards. They will be informed about the principle of voluntary participation and given the opportunity to withdraw at any time.

All information from the participants will be stored confidentially on a restricted research database (SAFE). Information about participants and contact information will be separated and a participant-ID will be used. Access to the database will be restricted to the researcher only. Paper-based documents will be shredded when the data is transferred to the SAFE hardware.

Ethical approvals are granted, and if we make any amendments to the protocol, we will notify the correct stakeholders. The National Center for Research Data (NSD/SIKT) approved our processing of personal data (reference 624,616). The Regional Ethics Committee assessed this project to be outside of their scope and hence we did not need a permit (reference 480,807).

## 5 Conclusion

To the best of our knowledge, this is the first study to assess the feasibility and experienced improved health of an intervention integrating highly educated migrants waiting for their authorization to practice their profession in Norway. This study will evaluate both the intervention’s feasibility and its effectiveness on health, as measured by improvements in self-rated health along with qualitative and other quantitative measures. If found to be feasible and beneficial, it could be further evaluated for effect, replicated, and developed into a future strategy to integrate highly educated migrants into a new society. The findings from this study can also be used in other high-income countries that have similar accreditation processes in which migrants have to wait for a long time for processing of accreditations. Since the core of this study is engaging in meaningful experiences at work, this study for health professionals can also be extended to other professional groups such as foreign teachers among others. In addition to the importance of the study itself, pilot protocol papers such as this one, are central for recognizing challenges that may arise during the study, identifying mitigating measures, and providing lessons for the future when planning and executing complex pilots.

## References

[1] SSB. Statistics Norway. How many immigrants are overqualified? (2022). Available at: https://www.ssb.no/arbeid-og-lonn/sysselsetting/artikler/hvor-mange-innvandrere-er-overkvalifisert (Accessed January 2, 2023).

[2] NOU. Time for action — The personnel in a sustainable health and care services Norwegian Ministry of Health and Care Services 2023. Ministry of Health and Welfare (2023). 4 p.

[3] SSB. Statistics Norway. Health care personnel 2014. Statistics Norway (2014).

[4] SSB. Statistics Norway. Immigrants and Norwegian-born to immigrant parents. Statistics Norway (2024).

[5] IMDI. The Norwegian directorate of integration and diversity. Immigrants in the labour market. The Norwegian directorate of integration and diversity (2023).

[6] Simons SM Rowland KN. Diversity and its impact on organizational performance: the influence of diversity constructions on expectations and outcomes. J Technol Manag Innov. (2011) 6:171–83. doi: 10.4067/S0718-27242011000300013

[7] Dobbin F Kalev A. Why diversity programs fail. Harv Bus Rev. (2016) 94:14.

[8] Dich N Lund R Hansen ÅM Rod NH. Mental and physical health effects of meaningful work and rewarding family responsibilities. PLoS One. (2019) 14:e0214916. doi: 10.1371/journal.pone.0214916, PMID: 31017925 PMC6481914

[9] Geurts N Davids T Spierings N. The lived experience of an integration paradox: why high-skilled migrants from Turkey experience little national belonging in the Netherlands. J Ethn Migr Stud. (2021) 47:69–87. doi: 10.1080/1369183X.2020.1770062

[10] Landolt S Thieme S. Highly skilled migrants entering the labour market: experiences and strategies in the contested field of overqualification and skills mismatch. Geoforum. (2018) 90:36–44. doi: 10.1016/j.geoforum.2018.01.009

[11] Anderson B Blinder S. Who counts as a migrant? Definitions and their consequences. Briefing, the migration Observatory at the University of Oxford. Centre on Migration, Policy and Society (COMPAS) University of Oxford (2011).

[12] Brell C Dustmann C Preston I. The labor market integration of refugee migrants in high-income countries. J Econ Perspect. (2020) 34:94–121. doi: 10.1257/jep.34.1.94

[13] Nowotny K. Are overqualified migrants self-selected? Evidence from central and eastern European countries. J Hum Cap. (2016) 10:303–46. doi: 10.1086/687415

[14] Povrzanović Frykman M Mozetič K. The importance of friends: social life challenges for foreign physicians in southern Sweden. Community Work Fam. (2020) 23:385–400. doi: 10.1080/13668803.2019.1599323

[15] Frykman MP Guribye E Hidle K Mozetič K. How does place matter to highly skilled migrants? Nord J Migr Res. (2020) 10:51–68. doi: 10.2478/njmr-2019-0026

[16] Przybyszewska A. Downward professional mobility among poles working and living in Norway. Nord J Migr Res. (2021) 11:35–49. doi: 10.33134/njmr.377

[17] Biavaschi C Burzyński M Elsner B Machado J. Taking the skill bias out of global migration. J Dev Econ. (2020) 142:102317. doi: 10.1016/j.jdeveco.2018.12.006

[18] Michel J-P Ecarnot F. The shortage of skilled workers in Europe: its impact on geriatric medicine. Eur Geriatr Med. (2020) 11:345–7. doi: 10.1007/s41999-020-00323-0, PMID: 32328964 PMC7176573

[19] World Health Organization. Transforming and scaling up health professionals’ education and training: World Health Organization guidelines 2013. Geneva, Switzerland: World Health Organization (2013). 26042324

[20] Walther L Rayes D Amann J Flick U Ta TMT Hahn E . Mental health and integration: a qualitative study on the struggles of recently arrived refugees in Germany. Front Public Health. (2021) 9:576481. doi: 10.3389/fpubh.2021.576481, PMID: 34805055 PMC8599120

[21] Moynihan D Herd P Gerinza J. Kafka’s bureaucracy: immigration administrative burdens in the trump era. Georgetown University, USA: Perspectives on Public Management and Governance (In Press). 10 p.

[22] Tibajev A Hellgren C. The effects of recognition of foreign education for newly arrived immigrants. Eur Sociol Rev. (2019) 35:506–21. doi: 10.1093/esr/jcz011

[23] Bygnes S. Not all Syrian doctors become taxi drivers: stagnation and continuity among highly educated Syrians in Norway. J Int Migr Integr. (2021) 22:33–46. doi: 10.1007/s12134-019-00717-5

[24] Sidorchuk A Engström K Johnson CM Leeoza NK Möller J. Employment status and psychological distress in a population-based cross-sectional study in Sweden: the impact of migration. BMJ Open. (2017) 7:e014698. doi: 10.1136/bmjopen-2016-014698, PMID: 28389494 PMC5558822

[25] Verkuyten M. The integration paradox: empiric evidence from the Netherlands. Am Behav Sci. (2016) 60:583–96. doi: 10.1177/0002764216632838, PMID: 27152028 PMC4827166

[26] Haj-Younes J Strømme EM Igland J Kumar B Abildsnes E Hasha W . Changes in self-rated health and quality of life among Syrian refugees migrating to Norway: a prospective longitudinal study. Int J Equity Health. (2020) 19:1–9. doi: 10.1186/s12939-020-01300-6 PMC7590794 33109202

[27] Haj-Younes J Strømme EM Igland J Abildsnes E Kumar B Hasha W . Health and healthcare access and utilization among Syrian refugees migrating to Norway: a longitudinal study. BMC Health Serv Res. (2022) 21:572. doi: 10.1186/s12913-021-06571-5 PMC8191125 34112164

[28] Pearson N Naylor P-J Ashe MC Fernandez M Yoong SL Wolfenden L. Guidance for conducting feasibility and pilot studies for implementation trials. Pilot Feasibility Stud. (2020) 6:1–12. doi: 10.1186/s40814-020-00634-w 33292770 PMC7603668

[29] Landes SJ McBain SA Curran GM. Reprint of: an introduction to effectiveness-implementation hybrid designs. Psychiatry Res. (2020) 283:112630. doi: 10.1016/j.psychres.2019.112630, PMID: 31722790

[30] Curran GM Bauer M Mittman B Pyne JM Stetler C. Effectiveness-implementation hybrid designs: combining elements of clinical effectiveness and implementation research to enhance public health impact. Med Care. (2012) 50:217–26. doi: 10.1097/MLR.0b013e3182408812, PMID: 22310560 PMC3731143

[31] Eisman AB Palinkas LA Brown S Lundahl L Kilbourne AM. A mixed methods investigation of implementation determinants for a school-based universal prevention intervention. Implement Res Pract. (2022) 3:26334895221124962. doi: 10.1177/26334895221124962, PMID: 37091102 PMC9978636

[32] Strømme EM Igland J Haj-Younes J Kumar BN Fadnes LT Hasha W . Chronic pain and mental health problems among Syrian refugees: associations, predictors and use of medication over time: a prospective cohort study. BMJ Open. (2021) 11:e046454. doi: 10.1136/bmjopen-2020-046454, PMID: 34548344 PMC8458374

[33] Larios DB Sam DL Sandal GM. Psychological distress among afghan refugees in Norway as a function of their integration. Front Psychol. (2023) 14:1143681. doi: 10.3389/fpsyg.2023.1143681 37143593 PMC10151542

[34] Guan M Han B. Factor structures of general health questionnaire-12 within the number of kins among the rural residents in China. Front Psychol. (2019) 10:1774. doi: 10.3389/fpsyg.2019.01774, PMID: 31428024 PMC6688627

[35] Lara-Cabrera ML Betancort M Muñoz-Rubilar A Rodríguez-Novo N Bjerkeset O Cuevas CDL. Psychometric properties of the WHO-5 well-being index among nurses during the COVID-19 pandemic: a cross-sectional study in three countries. Int J Environ Res Public Health. (2022) 19:10106. doi: 10.3390/ijerph191610106, PMID: 36011741 PMC9407690

[36] Eriksson M Lindström B. Validity of Antonovsky’s sense of coherence scale: a systematic review. J Epidemiol Community Health. (2005) 59:460–6. doi: 10.1136/jech.2003.018085, PMID: 15911640 PMC1757043

[37] Eriksson M Mittelmark MB. The sense of coherence and its measurement 12. In: Mittelmark MB, Bauer GF, Vaandrager L, Pelikan JM, Sagy S, Eriksson M. et al., editors. The handbook of salutogenesis. (2017). Springer Publishing Co, 97.

[38] Harder N Figueroa L Gillum RM Hangartner D Laitin DD Hainmueller J. Multidimensional measure of immigrant integration. Proc Natl Acad Sci. (2018) 115:11483–8. doi: 10.1073/pnas.1808793115, PMID: 30348786 PMC6233107

[39] NTNU. Norwegian University of Science and Technology. HUNT-3: the Nord-Trøndelag health study. Springer Publishing Co. (2023). Available at: https://www.ntnu.edu/hunt/hunt3 (Accessed January 2, 2023).

[40] del Pilar S-LM Dresch V. The 12-item general health questionnaire (GHQ-12): reliability, external validity and factor structure in the Spanish population. Psicothema. (2008) 20:839–43. 18940092

[41] Topp CW Østergaard SD Søndergaard S Bech P. The WHO-5 well-being index: a systematic review of the literature. Psychother Psychosom. (2015) 84:167–76. doi: 10.1159/000376585, PMID: 25831962

[42] Schmalbach B Zenger M Tibubos AN Kliem S Petrowski K Brähler E. Psychometric properties of two brief versions of the Hopkins symptom checklist: HSCL-5 and HSCL-10. Assessment. (2021) 28:617–31. doi: 10.1177/1073191119860910, PMID: 31272193

[43] Goldberg DP. User's guide to the general health questionnaire. London, England: Windsor (1988).

[44] World Health Organization. Wellbeing measures in primary health care/the DepCare project: Report on a WHO meeting: Stockholm, Sweden, 12–13 February 1998 World Health Organization. Stockholm, Sweden: Regional Office for Europe (1998).

[45] Braun V Clarke V. Thematic analysis American Psychological Association. Washington DC, USA (2012).

[46] Antonovsky A. The salutogenic model as a theory to guide health promotion. Health Promot Int. (1996) 11:11–8. doi: 10.1093/heapro/11.1.11

[47] Armat MR Assarroudi A Rad M. Inductive and deductive: ambiguous labels in qualitative content analysis. Qual Rep. (2018) 23:219–220. doi: 10.46743/2160-3715/2018.2872

[48] Tomasi J Warren C Kolodzey L Pinkney S Guerguerian A-M Kirsch R . Convergent parallel mixed-methods study to understand information exchange in paediatric critical care and inform the development of safety-enhancing interventions: a protocol study. BMJ Open. (2018) 8:e023691. doi: 10.1136/bmjopen-2018-023691, PMID: 30173162 PMC6120652

[49] Vittinghoff E Glidden DV Shiboski SC McCulloch CE. Regression methods in biostatistics: Linear, logistic, survival, and repeated measures models. Springer Publishing Co. (2005).

[50] Teresi JA Yu X Stewart AL Hays RD. Guidelines for designing and evaluating feasibility pilot studies. Med Care. (2022) 60:95–103. doi: 10.1097/MLR.0000000000001664, PMID: 34812790 PMC8849521

[51] Bowen DJ Kreuter M Spring B Cofta-Woerpel L Linnan L Weiner D . How we design feasibility studies. Am J Prev Med. (2009) 36:452–7. doi: 10.1016/j.amepre.2009.02.002, PMID: 19362699 PMC2859314

[52] Jong ST Croxson CH Foubister C Brown HE Guell C Lawlor ER . Reach, recruitment, dose, and intervention fidelity of the GoActive school-based physical activity intervention in the UK: a mixed-methods process evaluation. Children. (2020) 7:231. doi: 10.3390/children7110231, PMID: 33212854 PMC7698468

[53] Glass RL. Pilot studies: what, why and how. J Syst Softw. (1997) 36:85–97. doi: 10.1016/0164-1212(95)00197-2

