# Supplementary material for: Including highly educated migrants in academia to improve their health—protocol for a pilot intervention
Source: Front Public Health. 2024 Oct 28;12:1347992. doi: 10.3389/fpubh.2024.1347992 (PMC11551116; doi:10.3389/fpubh.2024.1347992)
Supplement: Supplementary file 1 [file Data_Sheet_1.pdf]

Supplementary material A: surveys norwegian/english

Dato:

Sted:

Helse gjennom meningsfulle integrerende yrkesrettede aktiviteter – En pilot intervensjonsstudie

Takk for at du deltar i denne studien!

Informasjonen i dette spørreskjemaet vil bli brukt i forskning rettet mot å forstå opplevelse av helse blant migranter med høyere helseutdanning i Norge. Det er viktig at du besvarer alle spørsmålene. Spør hvis det er noe du ikke forstår. Det utfylte spørreskjemaet skal gis tilbake til personene som inviterte deg til studien.

Svar ved å markere boksen (☐) eller besvare de åpne feltene ( ) som forklart i teksten.

Ved å besvare denne spørreundersøkelsen samtykker du til at vi bruker informasjonen kun til studiens formål. All informasjon behandles konfidensielt.

Hilsen Integrering for helse-gruppen,
Universitetet i Bergen

1. PERSONALIA

1.1 **Fornavn:**

Vennligst spesifiser

1.2 **Etternavn:**

Vennligst spesifiser

1.3 **Telefonnummer:**

Vennligst spesifiser

1.4 **Epostadresse:**

Vennligst spesifiser

2. BAKGRUNNSINFORMASJON

2.1 **Kjønn:**

☐ Mann ☐ Kvinne ☐ Annet

2.2 **Fødselsdato:**

__ . __ . ____ *(f.eks. 01.06.1978)*

2.3 **Fødeland:**

Vennligst spesifiser

2.4 **Ankomstår til Norge:** ____ *(f.eks. 2020)*

2.5 **Årsak til innvandring til Norge:**

☐ Arbeid ☐ Flukt/asyl ☐ Familiegjenforening
☐ Studier ☐ Annet

2.6 **Kryss av boksen som best beskriver ditt nivå av norskkunnskaper:**

A1	A2	B1	B2	C1	C2
☐	☐	☐	☐	☐	☐

2.7 Sivilstand:

- [ ] Gift
- [ ] Skilt
- [ ] Enke/enkemann
- [ ] Singel
- [ ] Samboende
- [ ] I et forhold

2.8 Hvor mange barn har du?

Ingen barn	1	2	3	4	5	6 eller flere
☐	☐	☐	☐	☐	☐	☐

2.9 Hvor mange er dere totalt i husstanden, inkludert deg selv?

1	2	3	4	5	6 eller flere
☐	☐	☐	☐	☐	☐

2.10 Bostedskommune:

Vennligst spesifiser

3. UTDANNING OG ARBEID

3.1 Hva jobbet du med før du kom til Norge?
(Du kan velge flere alternativer)

- [ ] Uten arbeid
- [ ] Militære yrker
- [ ] Lederyrker
- [ ] Akademiske yrker
- [ ] Kontoryrker
- [ ] Høyskoleyrker
- [ ] Bonde/fisker
- [ ] Salg- og serviceyrker
- [ ] Håndtverker
- [ ] Transportarbeider
- [ ] Renhold
- [ ] Prosess- og maskinoperatør
- [ ] Hjelpearbeider
- [ ] Annet

3.2 Hvilket alternativ beskriver best det du har gjort de siste fire ukene?
(Velg kun ett alternativ)

- [ ] Lønnet arbeid
- [ ] Hjelpearbeider/assistent
- [ ] Ulønnet arbeid (husarbeid, barnepass, e.l.)
- [ ] Arbeidsledig, aktivt jobbsøkende
- [ ] Arbeidsledig, ikke aktivt jobbsøkende
- [ ] Varig syk eller funksjonshemmet
- [ ] Pensjonist
- [ ] Skole
- [ ] Introduksjonsprogrammet/kvalifiseringsprogrammet
- [ ] Annet

3.3 Hva er din utdanningsbakgrunn?

- [ ] Tekniske og naturvitenskapelige fag
- [ ] Helsefag (medisin, sykepleie, farmasi e.l.)
- [ ] Samfunnsvitenskap, humaniora eller jus
- [ ] Yrkesfaglig retning
- [ ] Pedagogikk
- [ ] IT/media
- [ ] Annet

3.4 Hvis du har helsefaglig utdannelse, vennligst oppgi hva slags helseutdanning du har:

Vennligst spesifiser

3.5 Hvor mange år med skolegang har du fullført etter videregående skole?
(Inkludert høyere utdannelse)

☐☐ *(f.eks. 10)*

3.6 Hvilke(t) land er utdannelsen din fra?

Vennligst spesifiser

3.7 Hvilken grad kan utdannelsen din kategoriseres under i Norge?

- [ ] Bachelorgrad
- [ ] Mastergrad
- [ ] Doktorgrad
- [ ] Yrkesfaglig utdanning
- [ ] Ingen av disse

3.8 Har du en utdannelse du ikke har fullført, eller som har blitt avbrutt?

Ja	Nei
☐	☐

3.9 Hvis ja, innen hvilket fagområde?

- [ ] Tekniske og naturvitenskapelige fag
- [ ] Helsefag (medisin, sykepleie, farmasi e.l.)
- [ ] Samfunnsvitenskap, humaniora eller jus
- [ ] Yrkesfaglig retning
- [ ] Pedagogikk
- [ ] IT/media
- [ ] Annet

3.10 Er utdanningen din relevant for jobben din nå?
(F.eks. sykepleie for en person som jobber i helsevesenet)

Ja	Nei
☐	☐

3.11 Har du søkt om å få utdannelsen din godkjent i Norge?

Ja	Nei	Trenger ikke
☐	☐	☐

3.12 Hvis ja, hvor mye av utdannelsen må du ta på nytt?

☐ Ingen deler
☐ Hele utdanningen
☐ Kun noen fag
☐ Nesten hele utdanningen
☐ Vet ikke

4. HELSE, LIVSKVALITET OG FUNKSJON

4.1 Hvordan vurderer du alt i alt din egen helse?

Svært god	God	Verken god eller dårlig	Dårlig	Svært dårlig
☐	☐	☐	☐	☐

4.2 Lider du av langvarig (minst 1 års) sykdom eller skade av fysisk eller psykisk art som svekker ditt daglige liv?

Ja	Nei
☐	☐

4.3 Hvis ja, hvordan vil du beskrive svekkelsen?

	Lett	Moderat	Alvorlig
4.3.1 Motorisk svekkelse	☐	☐	☐
4.3.2 Nedsatt syn	☐	☐	☐
4.3.3 Nedsatt hørsel	☐	☐	☐
4.3.4 Svekkelse på grunn av fysisk sykdom	☐	☐	☐
4.3.5 Svekkelse på grunn av psykiske helseproblemer	☐	☐	☐

4.4 Hvor mange timer av dagen er du stillesittende?
(Både hjemme, på skolen og på jobb)

Omtrent ☐ timer *(f.eks. 6 timer)*

5. TRIVSEL OG VELVÆRE

4.1 Merk alternativet som best beskriver hvordan du har følt deg i løpet av de siste to ukene:
(Høyere tall betyr bedre velvære)

	Hele tiden					Aldri
5.1.1 Jeg har følt meg glad og i godt humør	5	4	3	2	1	0
5.1.2 Jeg har følt meg rolig og avslappet	5	4	3	2	1	0
5.1.3 Jeg har følt meg aktiv og sterk	5	4	3	2	1	0
5.1.4 Jeg har følt meg opplagt og uthvilt når jeg våkner	5	4	3	2	1	0
5.1.5 Jeg har følt at mitt daglige liv har vært fylt av ting som interesserer meg	5	4	3	2	1	0

6. GENERELLE HELSESPØRSMÅL

6.1 I løpet av de siste to ukene, har du:

	Bedre enn vanlig	Som vanlig	Mindre enn vanlig	Mye mindre enn vanlig
6.1.1 Vært i stand til å konsentrere deg om det du har drevet med?				
6.1.2 Mistet søvn på grunn av bekymringer?				
6.1.3 Følt at du tar del i ting på en nyttig måte?				
6.1.4 Følt at du er i stand til å ta beslutninger om ulike ting?				

	Bedre enn vanlig	Som vanlig	Mindre enn vanlig	Mye mindre enn vanlig
6.1.5 Følt deg stadig under press?				
6.1.6 Følt deg ute av stand til å mestre vanskeligheter?				
6.1.7 Vært i stand til å glede deg over dine daglige gjøremål?				
6.1.8 Vært i stand til å møte utfordringer?				
6.1.9 Mistet troen på deg selv?				
6.1.10 Følt deg ulykkelig eller deprimert?				
6.1.11 Tenkt på deg selv som en verdiløs person?				
6.1.12 Stort sett følt deg bra i hverdagen				

7. INTEGRERING

7.1 Hvor knyttet føler du deg til Norge?

- ☐ Jeg fløer en ekstremt nær forbindelse
- ☐ Jeg føler en veldig nær forbindelse
- ☐ Jeg føler en moderat tilknytning
- ☐ Jeg føler en svak tilknytning
- ☐ Jeg føler ikke en tilknytningi det hele tatt

7.2 Hvor ofte kjenner du på utenforskap i Norge?

Aldri	Sjelden	Noen ganger	Ofte	Alltid
☐	☐	☐	☐	☐

7.3 Når du tenker på fremtiden din, hvor vil du bo?

- ☐ Jeg vil definitivt bo i Norge resten av livet
- ☐ Jeg vil nok bo i Norge resten av livet
- ☐ Jeg er usikker på om jeg vil bli i Norge eller om jeg vil flytte til et annet land
- ☐ Jeg vil nok flytte til et annet land
- ☐ Jeg vil definitivt flytte til et annet land

7.4 Hvor ofte føler du deg isolert fra det norske samfunnet?

Aldri	Sjelden	Noen ganger	Ofte	Alltid
☐	☐	☐	☐	☐

7.5 Hvor vanskelig eller enkelt ville det være for deg finne jobb i Norge?

- ☐ Veldig vanskelig
- ☐ Litt vanskelig
- ☐ Verken vanskelig eller lett
- ☐ Litt lett
- ☐ Veldig lett

7.6 I løpet av <u>de siste 12 månedene</u>, hvor ofte har du spist med nordmenn som ikke er en del av din familie?

- ☐ Aldri
- ☐ En gang i året
- ☐ En gang i måneden
- ☐ En gang i uken
- ☐ Nesten hver dag

7.7 Tenk på nordmennene i telefonkontaktene dine eller blant kontaktene dine på sosiale medier. Hvor mange av dem hadde du en samtale med, enten via telefon, Messenger-chat eller tekstutveksling, i løpet av <u>de siste 4 ukene</u>?

☐ 0 ☐ 1–2 ☐ 3–6
☐ 7–14 ☐ 15 eller mer

7.8 Vennligst vurder dine egne ferdigheter i norsk. Hvor godt kan du gjøre følgende når du leser norsk? Jeg kan lese og forstå hovedpoengene i enkle avisartikler om kjente emner

- ☐ Veldig godt
- ☐ Godt
- ☐ Verken godt eller dårlig
- ☐ Dårlig
- ☐ Veldig dårlig

7.9 Vennligst vurder dine egne ferdigheter i norsk. Hvor godt kan du gjøre følgende når du snakker norsk? I en samtale kan jeg snakke om kjente temaer og uttrykke personlige meninger

- ☐ Veldig godt
- ☐ Godt
- ☐ Verken godt eller dårlig
- ☐ Dårlig
- ☐ Veldig dårlig

8. DISKRIMINERING

8.1 **Hvor ofte opplever du følgende situasjoner i Norge?**

	Aldri	Sjelden	Noen ganger	For det meste	Ofte	Veldig ofte
8.1.1 Diskriminering fra norske myndigheter	1	2	3	4	5	6
8.1.2 Diskriminering i skolen eller på jobben	1	2	3	4	5	6
8.1.3 Føler deg lite respektert på grunn av din nasjonale bakgrunn	1	2	3	4	5	6
8.1.4 Folk som kommer med rasistiske ytringer mot deg	1	2	3	4	5	6

9. PSYKISK HELSE

9.1 **Angi hvor mye du har vært plaget av de følgende symptomene i løpet av den siste uken.**

	Ikke plaget i det hele tatt	Litt plaget	Ganske plaget	Veldig plaget
9.1.1 Plutselig frykt uten grunn	1	2	3	4
9.1.2 Følt deg redd	1	2	3	4
9.1.3 Besvimelse, svimmelhet eller svakhet	1	2	3	4
9.1.4 Følt deg anspent eller sliten	1	2	3	4
9.1.5 Klandret deg selv for ting	1	2	3	4
9.1.6 Vanskeligheter med å sovne	1	2	3	4
9.1.7 Følt deg trist	1	2	3	4
9.1.8 Følt deg verdiløs	1	2	3	4
9.1.9 Følt at alt er et slit	1	2	3	4
9.1.10 Følt på håpløshet med hensyn til fremtiden	1	2	3	4

10. OPPLEVELSE AV SAMMENHENG

10.1 **Her er en rekke spørsmål knyttet til ulike aspekter av livet ditt. Marker tallet som best uttrykker ditt svar.**
(Kun ett svar per spørsmål)

	Veldig sjelden eller aldri						Veldig ofte
10.1.1 Føler du i bunn og grunn at du ikke bryr deg om hva som skjer rundt deg?	1	2	3	4	5	6	7

	Aldri						Alltid
10.1.2 Har det hendt at du ble overrasket over oppførselen til personer som du trodde du kjente godt?	1	2	3	4	5	6	7

	Aldri						Alltid
10.1.3 Har det hendt at du ble skuffet av personer som du har stolt på?	1	2	3	4	5	6	7

	Ingen klare mål eller hensikt						Svært klare mål og hensikt
10.1.4 Inntil nå har livet ditt hatt:	1	2	3	4	5	6	7

	Veldig ofte						Veldig sjelden eller aldri
10.1.5 Har du følt at du blir urettferdig behandlet?	1	2	3	4	5	6	7

	Veldig ofte						Veldig sjelden eller aldri
10.1.6 Har du opplevd å være i en ukjent situasjon der du ikke vet hva du skal gjøre?	1	2	3	4	5	6	7

	En kilde til dyp glede og tilfredshet						En kilde til smerte og kjedsomhet
10.1.7 Å gjøre det du gjør hver dag er:	1	2	3	4	5	6	7

	Veldig ofte						Veldig sjelden eller aldri
10.1.8 Har du veldig uklare følelser og tanker?	1	2	3	4	5	6	7

	Veldig ofte						Veldig sjelden eller aldri
10.1.9 Hender det at du har følelser inni deg som du ikke ønsker å ha?	1	2	3	4	5	6	7

	Aldri						Veldig ofte
10.1.10 Mange mennesker, selv karaktersterke, føler seg noen ganger som tapere i visse situasjoner. Hvor ofte har du følt det slik?	1	2	3	4	5	6	7

	Du over- eller undervurderte betydningen						Du så ting i riktig proporsjon
10.1.11 Når noe har hendt, har du generelt opplevd at:	1	2	3	4	5	6	7

	Veldig ofte						Veldig sjelden eller aldri
10.1.12 Hvor ofte føler du at det er liten mening i de tingene du gjør daglig?	1	2	3	4	5	6	7

	Veldig ofte						Veldig sjelden
10.1.13 Hvor ofte har du følelser som du ikke er sikker på at du kan holde under kontroll?	1	2	3	4	5	6	7

11. OPPFØLGING

	Ja	Nei
**11.1 Til slutt vil vi vite om du samtykker til å bli kontaktet igjen i forbindelse med studien etter seks måneder for å gjennomføre en ny spørreundersøkelse. Det er viktig for oss å vite hvordan det går med din helse.**	☐	☐

TAKK FOR AT DU SVARER PÅ DISSE SPØRSMÅLENE! HUSK Å RETURNERE DETTE SKJEMAET TIL PERSONEN SOM GAV DEG DET FØR DU DRAR.

Date:

Place:

Health through meaningful integrative occupational activities for highly educated migrants – a pilot intervention study

Thank you for taking part in this study!

The information in this questionnaire will be used in research aimed at understanding the experience of health among migrants with higher health-related education in Norway. It is important that you answer all the questions. Ask if there is something you don't understand. The completed questionnaire must be returned to the people responsible for the study.

Please answer by putting an X in the box (☐), or answer the open fields (   ) as explained in the text.

By answering this survey, you agree that we use the information only for the purpose of the study. All information will be treated confidentially.

The Integration for health-group,
The University of Bergen

1. PERSONALIA

1.1 **First name:**

Please specify

1.2 **Last name:**

Please specify

1.3 **Phone:**

Please specify

1.4 **Email address:**

Please specify

2. BACKGROUND INFORMATION

2.1 **Gender:**

☐ Male ☐ Female ☐ Other

2.2 **Date of birth:**

☐☐ . ☐☐ . ☐☐☐☐ *(e.g. 01.06.1978)*

2.3 **Country of birth:**

Please specify

2.4 **Year of arrival to Norway:** ☐☐☐☐ *(e.g. 2020)*

2.5 **Reason for emigrating to Norway:**

☐ Work ☐ Refuge/asylum ☐ Family reunification

☐ Studies ☐ Other

2.6 **Tick the box that best describes your level of proficiency in Norwegian:**

A1	A2	B1	B2	C1	C2
☐	☐	☐	☐	☐	☐

2.7 Marital status:

- [ ] Married
- [ ] Divorced
- [ ] Widow/widower
- [ ] Single
- [ ] Cohabiting
- [ ] In a relationship

2.8 How many children do you have?

No children	1	2	3	4	5	6 or more
☐	☐	☐	☐	☐	☐	☐

2.9 How many people, including yourself, live in your household?

1	2	3	4	5	6 or more
☐	☐	☐	☐	☐	☐

2.10 Municipality of residence:

Please specify

3. EDUCATION AND EMPLOYMENT

3.1 In what field did you work before you arrived in Norway?
(You may select more than one option)

- [ ] I did not work
- [ ] Leadership
- [ ] Office work
- [ ] Farming/fishing
- [ ] Craftship
- [ ] Cleaning
- [ ] Helper
- [ ] Military occupations
- [ ] Academic occupations
- [ ] University college occupations
- [ ] Sales and service occupations
- [ ] Transportation
- [ ] Process and machine operators
- [ ] Other

3.2 Which of the following options best describes what you have been doing for <u>the last four weeks</u>?
(Please select only one)

- [ ] Paid employment
- [ ] Helper/assistant
- [ ] Unpaid labour (household chores, childcare, or similar)
- [ ] Unemployed, actively looking for a job
- [ ] Unemployed, not actively looking for a job
- [ ] Permanently sick or disabled
- [ ] Retired
- [ ] In school
- [ ] Introduction program/qualification program
- [ ] Other

3.3 In what field is your educational background?

- [ ] Natural sciences and technical subjects
- [ ] Health sciences (medicine, nursing, pharmacy, or similar)
- [ ] Social sciences, humanities or law
- [ ] Vocational specialization
- [ ] Pedagogy
- [ ] IT/media
- [ ] Other

3.4 If you have a health professional background, please specify your health professional background:

Please specify

3.5 How many years of schooling have you completed after high school?
(Including higher education)

(e.g. 10)

3.6 Which country/countries is your education from?

Please specify

3.7 What degree can your education level be categorized as in Norway?

- [ ] Bachelor's degree
- [ ] Master's degree
- [ ] Doctorate (Ph.D.)
- [ ] Vocational education
- [ ] None of the above

	Yes	No
**3.8 Do you have any incomplete or interrupted degrees/studies?**	☐	☐

3.9 If yes, within which field?

- [ ] Natural sciences and technical subjects
- [ ] Health sciences (medicine, nursing, pharmacy, or similar)
- [ ] Social sciences, humanities or law
- [ ] Vocational specialization
- [ ] Pedagogy
- [ ] IT/media
- [ ] Other

3.10 Is your educational background relevant to your current job?
(E.g. nursing for someone employed within health care)

Yes ☐ No ☐

3.11 Have you applied for approval of your education in Norway?

Yes ☐ No ☐ No need ☐

3.12 If yes: How much of your education did you have to redo/repeat?

☐ Nothing ☐ The entire education
☐ Some subjects ☐ Almost the entire education
☐ Don't know

4. HEALTH, FUNCTION LEVEL AND QUALITY OF LIFE

4.1 How do you consider your health at the moment?

Very good	Good	Neither	Poor	Very poor
☐	☐	☐	☐	☐

4.2 Do you suffer from long-term (at least 1 year) illness or injury of a physical or psychological nature that impairs your daily life?

Yes ☐ No ☐

4.3 If yes, how would you describe your impairment?

	Slight	Moderate	Severe
4.3.1 Motor ability impairment	☐	☐	☐
4.3.2 Vision impairment	☐	☐	☐
4.3.3 Hearing impairment	☐	☐	☐
4.3.4 Impairment due to physical illness	☐	☐	☐
4.3.5 Impairment due to mental health problems	☐	☐	☐

4.4 How many hours do you approximately sit during a normal day?
(Including both work hours and leisure time)

About ☐ hours *(e.g. 6 hours)*

5. WELL-BEING

5.1 Please respond to each of the statements below by marking the option that best describes how you have felt during the last two weeks:

	All of the time					Never
5.1.1 I have felt cheerful and in good spirits	5	4	3	2	1	0
5.1.2 I have felt calm and relaxed	5	4	3	2	1	0
5.1.3 I have felt active and vigorous	5	4	3	2	1	0
5.1.4 I have woken up feeling fresh and rested	5	4	3	2	1	0
5.1.5 My daily life has been filled with things that interest me	5	4	3	2	1	0

6. GENERAL HEALTH QUESTIONNAIRE

6.1 During the past two weeks, have you:

6.1.1 Been able to concentrate on what you have been doing?	Better than usual	As usual	Less than usual	A lot less than usual
6.1.2 Lost much sleep over worry?	Has not happened	Not more than usual	More than usual	I slept a lot less than usual
6.1.3 Felt that you are playing a useful part in things?	More than usual	As usual	Less than usual	A lot less than usual
6.1.4 Felt capable of making decisions about things?	More than usual	As usual	Less than usual	A lot less than usual

6.1.5 Felt constantly under strain?	Not at all	Not more than usual	More than usual	A lot more than usual
6.1.6 Felt you couldn't overcome your difficulties?	Not at all	Not more than usual	More than usual	A lot more than usual
6.1.7 Been able to enjoy your normal day to day activites?	More than usual	As usual	Less than usual	A lot less than usual
6.1.8 Been able to face up to your problems?	Better than usual	As usual	Less than usual	A lot less than usual
6.1.9 Been losing confidence in yourself?	Not at all	Not more than usual	More than usual	A lot more than usual
6.1.10 Been feeling unhappy or depressed?	Not at all	Not more than usual	More than usual	A lot more than usual
6.1.11 Been thinking about yourself as a worthless person?	Not at all	Not more than usual	More than usual	A lot more than usual
6.1.12 Been feeling reasonably happy for day-to-day activities?	More than usual	As usual	Less than usual	A lot less than usual

7. INTEGRATION

7.1 How connected do you feel with Norway?

- ☐ I feel an extremely close connection
- ☐ I feel a very close connection
- ☐ I feel a moderately close connection
- ☐ I feel a weak connection
- ☐ I do not feel a connection at all

7.2 How often do you feel like an outsider in Norway?

Never	Rarely	Some times	Often	Always
☐	☐	☐	☐	☐

7.3 When you think about your future, where do you want to live?

- ☐ I will definitely live in Norway for the rest of my life
- ☐ I will probably live in Norway for the rest of my life
- ☐ I am not sure whether I want to stay in Norway or move to another country
- ☐ I will probably move to another country
- ☐ I will definitely move to another country

7.4 How often do you feel isolated from the Norwegian society?

Never	Rarely	Some times	Often	Always
☐	☐	☐	☐	☐

7.5 How difficult or easy would it be for you to find a job in Norway?

- ☐ Very difficult
- ☐ Somewhat difficult
- ☐ Neither difficult nor easy
- ☐ Somewhat easy
- ☐ Very easy

7.6 In <u>the last 12 months</u>, how often did you eat dinner with Norwegians who are not part of your family?

- ☐ Never
- ☐ Once a year
- ☐ Once a month
- ☐ Once a week
- ☐ Almost every day

7.7 Please think about the Norwegians in your phone contacts or among your social media contacts. With how many of them did you have a conversation - either by phone, messenger chat, or text exchange, in <u>the last 4 weeks</u>?

- ☐ 0
- ☐ 1–2
- ☐ 3–6
- ☐ 7–14
- ☐ 15 or more

7.8 Please evaluate your own skills in Norwegian. How well can you do the following when reading Norwegian? I can read and understand the main points in simple newspaper articles on familiar subjects:

- ☐ Very well
- ☐ Well
- ☐ Moderately well
- ☐ Not well
- ☐ Not well at all

7.9 Please evaluate your own skills in Norwegian. How well can you do the following when speaking Norwegian? In a conversation, I can speak about familiar topics and express personal opinions:

- ☐ Very well
- ☐ Well
- ☐ Moderately well
- ☐ Not well
- ☐ Not well at all

8. DISCRIMINATION

8.1 **How often do you experience the following situations in Norway?**

	Never	Seldom	Some times	Most of the time	Often	Very often
8.1.1 Discrimination by Norwegian authorities	1	2	3	4	5	6
8.1.2 Discrimination in school or at work	1	2	3	4	5	6
8.1.3 Feeling disrespected due to your national background	1	2	3	4	5	6
8.1.4 People making racist remarks towards you	1	2	3	4	5	6

9. MENTAL HEALTH

9.1 **Please mark the option that best describes how much each of the following feelings has bothered you during the last week.**

	Not at all	A little	Somewhat	Very much
9.1.1 Suddenly scared for no reason	1	2	3	4
9.1.2 Felt scared	1	2	3	4
9.1.3 Fainting, dizziness or weakness	1	2	3	4
9.1.4 Felt tense or tired	1	2	3	4
9.1.5 Blamed yourself for things	1	2	3	4
9.1.6 Difficulty falling or staying asleep	1	2	3	4
9.1.7 Felt sad	1	2	3	4
9.1.8 Felt worthless	1	2	3	4
9.1.9 Felt that everything is an effort	1	2	3	4
9.1.10 Felt hopeless about the future	1	2	3	4

10. SENSE OF COHERENCE

10.1 **Here is a series of questions relating to various aspects of your life. Each question has seven possible answers. Please mark the number the best expresses your answer.**
Please give only one answer to each question

	Very seldom or never						Very often
10.1.1 Do you have the feeling that you don't really care about what goes on around you?	1	2	3	4	5	6	7

	Never happened						Always happened
10.1.2 Has it happened in the past that you were surprised by the behaviour of people you thought you knew well?	1	2	3	4	5	6	7

	Never happened						Always happened
10.1.3 Has it happened that people whom you counted on disappointed you?	1	2	3	4	5	6	7

	No clear goals or purpose at all						Very clear goals and purpose
10.1.4 Until now your life has had:	1	2	3	4	5	6	7
	Very often						Very seldom or never
10.1.5 Do you have the feeling that you're being treated unfairly?	1	2	3	4	5	6	7
	Very often						Very seldom or never
10.1.6 Do you have the feeling that you are in an unfamiliar situation and don't know what to do?	1	2	3	4	5	6	7
	A source of deep pleasure and satisfaction						A source of pain and boredom
10.1.7 Doing the thing you do every day is:	1	2	3	4	5	6	7
	Very often						Very seldom or never
10.1.8 Do you have very mixed-up feelings and ideas?	1	2	3	4	5	6	7
	Very often						Very seldom or never
10.1.9 Does it happen that you have feelings inside you would rather not feel?	1	2	3	4	5	6	7
	Never						Very often
10.1.10 Many people – even those with a strong character – sometimes feel like sad sacks (losers) in certain situations. How often have you felt this way in the past?	1	2	3	4	5	6	7
	You overestimated or underestimated its importance						You saw things in the right proportion
10.1.11 When something happened, have you generally found that:	1	2	3	4	5	6	7
	Very often						Very seldom or never
10.1.12 How often do you have the feeling that there's little meaning in the things you do in your daily life?	1	2	3	4	5	6	7
	Very often						Very seldom
10.1.13 How often do you have feelings that you're not sure you can keep under control?	1	2	3	4	5	6	7

11. FOLLOW-UP

	Yes	No
11.1 **Finally, we would like to ask you for your permission to contact you again for the project after six months to conduct a new survey. It is important for us to know how you are doing in terms of health.**	☐	☐

THANK YOU FOR ANSWERING THESE QUESTIONS! PLEASE MAKE SURE TO RETURN THIS FORM TO THE PERSON WHO GAVE IT TO YOU BEFORE LEAVING.

Supplementary Material

Supplementary material B: interview guides for participants and mentors along with consent forms for both.

Interview guide for mentors

The purpose of the interview is to learn more about how the mentors experienced having participants present at their institute/research group.

The mentor role

• What has it been like to have the participant at your research group?

• How did you personally experience having a participant with you?

• What types of tasks were performed? What worked/what was good?

• What was the challenge of being a mentor?

Perceived effect

• How do you think the participants' presence has affected the academic environment at your research group?

• How do you think the participants' presence at the professional environment has affected the students?

• How do you think the participants' presence at the research group/institute has influenced you?

• How do you think the participants' presence at the research group/institute has affected the participants themselves?

Final question:

• -How can this mentoring scheme be improved?

Any final input?

- Thank you for participating!

UNIVERSITY OF BERGEN

Health for integration

The purpose of the interview is to learn more about how the participants experienced the intervention and how it has affected their health.

In-depth interview guide for participants

Participation in the intervention
What impressions are you left with after the intervention?
How was the planning and implementation of the course?
What was the biggest challenge of being a participant in the intervention?
- What worked?
- What did not work?
-What dividend are you left with?
How did you experience the match with your mentor?
To what extent did you use your own professional and multicultural competence?
-Did any challenges or disagreements arise?
-How was the division of tasks between you and the mentor?
-How did the communication with the students and the dissemination of subjects / competence go?
How do you think the implementation of the modules went? Anything you missed?

Effect on health
To what extent has the absence of relevant work and long processing time of educational papers affected your health?
In what way has the intervention and participation in the educational institutions affected your health?
In what way has the intervention affected your family's health?

Perceived effect on diversity
How do you think your participation in the intervention and presence has affected the academic environment / department/institute?
How do you think your presence at your department / faculty has affected the students?
How could the program have been better adapted to minorities with higher education?

Lastly:
-How can this mentor-program be made more accurate and effective? Thoughts on improvement?
- Any last input?
Thank you for participating!

Consent form and invitation letter to participate in the study:

"Health through meaningful integrative occupational activities for highly educated immigrants - a pilot intervention study"

Background information: purpose of the study

The University of Bergen invites you to participate in a pilot intervention study that will enable highly educated migrants with a health professional background that has not been approved of in Norway, to participate in teaching activities at UiB and HVL. The pilot study is a part of a PhD-thesis.
The intervention will measure the effect of meaningful integration on health among migrants and their families. Our understanding of the term meaningful integration entails that the participants will use their own expertise as much as possible. Furthermore, we also want to investigate how the presence of migrants at educational institutions contributes to increased cultural awareness and cultural competence among staff and students.
The research results will contribute to an increased knowledge base about meaningful integration among highly educated migrants in Norway. This is an invitation letter and a consent form to participate in the study.

Who oversees the research project?

Project manager: Dr. *Esperanza Diaz* and PhD-student *Khadra Yasien Ahmed (46907322).* You can contact us for more information as well.

The intervention has the following steps:

- Obtain consent forms so participants can conduct the first survey
- Background check/assessment and invitation to participate in the intervention
- Participating in a kick-off day and two additional preparation days
- Matching participants with a professional environment and mentor at UIB/HVL
- Placement at the educational institutions UIB/HVL
- Midway assessment through interviews
- Final interviews and second survey to be conducted

Who can participate in the study?

- Migrant background with legal residence
- Health professional background that is not approved in Norway (educated outside Norway)
- Not in relevant work despite higher education
- Norwegian level A2-B1
- Residing in Bergen and the surrounding area
- Desire to participate for six months

What does the intervention entail?

After assessing your background, you will be assigned to a professional environment and a mentor. You will collaborate with the environment for teaching students for one semester. The assignments you receive will be chosen in dialogue between you and the professional group you will be part of. It is expected that you have some individual teaching, but also that you participate in other coworkers' teachings. Participation in the intervention can be combined with the mandatory tasks you have if you attend the introductory program or similar programs under the auspices of the municipality. You will receive proof that you have participated in the intervention, with a more detailed description of your tasks when the intervention ends.

Right to withdraw and refuse participation

Participation is voluntary. You don't have to participate if you don't want to. You can withdraw at any time during the study without giving any reason. All information about you will then be deleted. When the research project ends on 30.04.2027, all data will be anonymized. This study nor the researchers are connected to any legal institution or the state administration and cannot influence the approval of your educational papers.

Your privacy - how we store and use your information

We will not share your information with others. Only the researchers in the study have access to the information and unauthorized have no access. We will replace your name and contact details with a

code that is stored on a separate name list separate from other data.
We store all information on a secure computer.
We delete audio recordings from the interview when we have written down everything we have talked about.
We make sure that no one can recognize you when we write research articles. For example, we will invent a different name when we write about you.
We adhere to the laws on privacy.

Your rights

If information about you appears in what we write, or have in our documents, you have the right to see what information about you we collect. You can also request that the information be deleted so that it no longer exists. If there is any information that is incorrect, you can report it and ask the researcher to correct it. You can also ask to receive a copy of the information from us. You can also complain to the Norwegian Data Protection Authority if you think that we have processed the information about you in a careless way or in a way that is not correct.
If you have questions related to the Data protections' assessment of the project, you can contact this service at: personverntjenester@sikt.no or 53 21 15 00

What gives us the right to process your personal data?

We process information about you based on your consent. On behalf of the University of Bergen, Personal Protection Services has assessed that the processing of personal data in this project is in accordance with the privacy regulations.

Consent for participation in the study:

I have read the information, or it has been read to me. I have had the opportunity to ask questions about it and any questions that I have asked, have been answered. I consent voluntarily to partake in the study, conduct the questionnaire twice and be interviewed twice. I know that I can refuse to participate at any time without having to explain why.

Date:

Participant's signature Researcher's signature

Invitation to participate as a mentor in a study

"Health through meaningful integrative occupational activities for highly educated immigrants - a pilot intervention study"

Background information

The University of Bergen invites you to partake in a pilot intervention study as a mentor. This pilot study will enable highly educated migrants with a health professional background that has not been approved of in Norway, to participate in teaching activities at UiB and HVL. The pilot study is a part of an PhD-thesis.
The intervention will measure the effect of meaningful integration on health among migrants and their families. Our understanding of the term meaningful integration entails that the participants will use their own expertise as much as possible. Furthermore, we also want to investigate how the presence of migrants at educational institutions contributes to increased cultural awareness and cultural competence among staff and students.
The results of the research will contribute to an increased knowledge base about meaningful integration among highly educated migrants in Norway. This is an invitation and consent letter to participate in the study as a mentor and be interviewed.

Who oversees the research project?

Project manager: Dr. *Esperanza Diaz* and PhD student *Khadra Yasien Ahmed (46907322)* that you also can contact for further information

Who are we looking for and what does participation entail?

- Academic staff at HVL or UIB

- Willing to be the main contact person for one or more migrants during deployment
- Willing to instruct and include migrants in their own work
- Desire to participate/commit for six months, preferably longer

The intervention has the following steps for mentors:

- Obtain consent forms from mentors to partake in the study
- Matching mentor and the professional group with the right participants
- Final interviews with mentors

Right to withdraw and refuse participation

Participation is voluntary. You don't have to participate if you don't want to. You can withdraw at any time during the study without giving any reason. All information about you will then be deleted. We store your data properly and in accordance with the Personal Protection Act.

As long as you can be identified in the data material, you have the right to:

· access to the information we process about you, and to be given a copy of the information

· to have information about you corrected that is incorrect or misleading

· to have personal data about you deleted

· to send a complaint to the Norwegian Data Protection Authority about the processing of your personal data

All data is stored securely on a secure computer and anonymized after the project's expiry date, 30.04.2027

If you have questions related to the Data protections' assessment of the project, you can contact this service at: personverntjenester@sikt.no or 53 21 15 00

What gives us the right to process your personal data?

We process information about you based on your consent. On behalf of the University of Bergen, Personal Protection Services has assessed that the processing of personal data in this project is in accordance with the privacy regulations.

Consent for participation in the study:

I have read the information, or it has been read to me. I have had the opportunity to ask questions about it and any questions that I have asked, have been answered. I consent voluntarily to partake in the study and be interviewed. I know that I can refuse to participate at any time without having to explain why. **Date:**

Mentor's signature **Researchers signature:**

Supplementary Material c:

Midterm evaluation scheme for participants

-Observere interaksjon, språkbruk, arbeidsfordeling av oppgaver og generell integrering

Spørsmål til underveisevaluering for deltagere

Hensikten med intervjuet er å lære mer om hvordan deltakerne opplever intervensjonen midtveis for å gjøre nødvendige endringer i tide for å forbedre resultatet

1. Hva har du gjort så langt i intervensjonen?
2. Hvordan føler kursdagene har forberedt deg for utplassering?
3. Hvordan har planleggingen med mentor gått? Hvordan har du bidratt direkte og indirekte?
4. Hvordan er det ved instituttet/faggruppen du er tilknyttet?
5. Hvordan har det vært å undervise på norsk?
6. Hvordan er det å delta på undervisning av norske studenter?
7. Hvordan har intervensjonen påvirket din og din families helse så langt?
8. Hvilke utfordringer har oppstått og hvordan har du løst dem?

Med mentor
Med instituttet/faggruppen

Questions to the midway- evaluation for participants

The purpose of the interview is to learn more about how the participants are experiencing the intervention midway to make the necessary changes as to improve the end-result

Observe interaction, language use, tasks preformed, division of tasks and general integration.

1. What have you done so far in the intervention?
2. In what way did the two course days prepare you for the placement at UIB-HVL?
3. How was the planning the teaching with the mentor? How have you contributed directly and indirectly?
4. How is it at the department / research group you are affiliated with?
5. How has it been to teach in Norwegian?
6. What is it like to participate in teaching Norwegian students?
7. How has the intervention affected your and your family's health so far?
8. What challenges have arisen and how have you solved them?

With mentor
With the department / research group/institute
